# Effects of vagus nerve stimulation on cognitive function in patients with epilepsy: a systematic review and meta-analysis

**DOI:** 10.3389/fneur.2024.1332882

**Published:** 2024-02-09

**Authors:** Yurou Kong, Kun Zhao, Duchun Zeng, Feiao Lu, Xiang Li, Yulun Wu, Zengming Jiang, Wanshun Wen

**Affiliations:** ^1^Center for Rehabilitation Medicine, Rehabilitation and Sports Medicine Research Institute of Zhejiang Province, Department of Rehabilitation Medicine, Zhejiang Provincial People’s Hospital (Affiliated People’s Hospital), Hangzhou Medical College, Hangzhou, Zhejiang, China; ^2^School of Medicine, Xiang'an Hospital of Xiamen University, Xiamen University, Xiamen, China

**Keywords:** vagus nerve stimulation, epilepsy, cognitive function, meta-analysis, review

## Abstract

**Background:**

Previous studies showed that vagus nerve stimulation (VNS) can improve cognitive function in patients with epilepsy, but there is still great controversy about the effect of VNS on cognitive function in patients with epilepsy.

**Objective:**

To investigate the effect of VNS on the cognitive function of epilepsy patients.

**Methods:**

Clinical trials published in PubMed, The Cochrane Library, and Embase before September 20, 2022, were comprehensively searched. Primary outcomes were overall cognitive performance, executive function, attention, memory; Secondary outcomes were seizure frequency, mood, and quality of life (QOL). Random effects were used to calculate the pooled outcome.

**Results:**

Twenty clinical trials were included. There was no significant improvement in overall cognitive performance in patients with epilepsy after VNS treatment (SMD = 0.07; 95% CI: −0.12 to 0.26; I^2^ = 0.00%) compared to pre-treatment. Compared to pre-treatment, there was no significant difference in executive function (SMD = −0.50; 95% CI: −1.50 to 0.50; *p* = 0.32), attention (SMD = −0.17; 95% CI: −0.43 to 0.09; *p* = 0.21) and memory (SMD = 0.64; 95% CI: −0.11 to 1.39; *p* = 0.09), but there were significant differences in seizure frequency, mood, and quality of life in patients with epilepsy after VNS.

**Conclusion:**

This meta-analysis did not establish that VNS can significantly improve cognitive function in patients with epilepsy, but it shows that VNS can significantly improve the seizure frequency, mood and quality of life of patients with epilepsy.

**Systematic review registration:**

https://www.crd.york.ac.uk/prospero/, identifier: CRD42023384059.

## 1 Introduction

Epilepsy is a chronic neurological disease characterized by recurrent and sudden abnormal discharges of brain neurons, which can lead to changes in motor function, sensory function, consciousness and behavioral function, presenting the characteristics of repetition, stereotype, transience and seizure (1). According to statistics, the global prevalence of epilepsy is between 0.5 and 1.0%, with over 50 million people affected, 80% of whom living in low- and middle-income countries (2, 3). Long-term frequent seizures and the side effects of anti-epileptic drugs will seriously damage the brain cell function of patients, affecting not only motor function, but also cognitive functions such as attention and memory. Even if the seizures are effectively controlled, the cognitive function impairment caused by the seizures cannot be reversed (4). It has been reported that more than 80% of patients with epilepsy are accompanied by varying degrees of cognitive decline, which seriously affects the QOL of patients and increases the economic burden on families and society (5).

Common treatments for epilepsy include medication, surgery, ketogenic diet, and neuromodulation techniques, of which VNS is the most commonly used neuromodulation technique. VNS has become an important means in the treatment of refractory epilepsy and has shown good clinical effects due to its advantages of no craniotomy, little trauma, wide surgical indications, good surgical safety and few complications (6). Previous studies have shown that VNS is effective in reducing seizure frequency in patients with epilepsy, and its efficacy has been widely recognized in several countries and regions (7–11). Although the main outcome indicator of the relevant clinical studies was the effect of VNS on seizures, some of these studies also reported its effect on cognitive function in patients with epilepsy. Studies have reported that the decrease in seizure frequency after VNS is often accompanied by an improvement in cognitive function, but there is no positive correlation between them, suggesting that the improvement in cognitive function may be due to VNS. However, the heterogeneity among the study results was high.

Therefore, the effect of VNS on cognitive function in patients with epilepsy remains highly controversial and no relevant meta-analysis has been published. In this study, we conducted a literature review and quantitative analysis of relevant published clinical trials to clarify the effects of VNS on cognitive function in patients with epilepsy and to provide direction and basis for future clinical research and treatment.

## 2 Materials and methods

A literature review and meta-analysis were conducted in accordance with the Cochrane Collaboration’s Preferred Reporting Items for Systematic Reviews and Meta-Analyses (PRISMA) Statement (12). This review followed a pre-registered protocol in PROSPERO (CRD42022384059).

### 2.1 Literature search

On September 20, 2022, a comprehensive search in PubMed, the Cochrane Library, and Embase for publications on the effects of VNS on cognitive function in patients with epilepsy was conducted. In addition, published reviews and references of included studies were searched for relevant studies. The search was performed using “epilepsy” and “vagus nerve stimulation” as keywords or Boolean terms. The complete search strategy can be found in Supplementary material.

### 2.2 Eligibility criteria

The inclusion criteria were: (1) population: patients diagnosed with epilepsy by EEG and clinical examination; (2) intervention: the treatment group was treated with VNS for a period of time, and the control group was treated with VNS before or when it was turned off; (3) outcomes: primary outcome was cognitive function, including overall cognitive performance or its various dimensions (executive function, attention, memory); secondary outcomes were seizure frequency, mood, and QOL. The study will be included in the meta-analysis regardless of the scale used to evaluate the outcome measures. The exclusion criteria were: (1) non-English literature; (2) review or opinion studies; (3) studies with incomplete data or inability to extract information; (4) duplicate published studies.

### 2.3 Study selection and data extraction

Each study was screened, extracted and cross-checked by 2 researchers independently. Discrepancies were resolved by discussion or consultation with a third party. Both 2 researchers and a third party in this study have clinical experience in the application of VNS for patients with epilepsy. Data extraction was conducted using a standardized form with specific items including study characteristics (author, year, country, sample size), subject characteristics (age, gender, type of epilepsy), VNS parameters (type of stimulation, stimulation site, pulse width, frequency, amplitude, on/off-time, duration), outcomes, and time of assessment.

### 2.4 Bias and quality assessment

The bias of each study was evaluated by 2 researchers independently and the results were cross-checked. Discrepancies were resolved by discussion or consultation with a third party. Randomized controlled studies were evaluated using the Cochrane Collaboration’s tool (13) and non-randomized controlled studies were evaluated using the methodological index for non-randomized studies (MINORS) (14).

### 2.5 Statistical analysis

Statistical analyses were performed using Stata 17.0 software. The outcome measures were all continuous variables, so mean difference (MD) was used as the effect indicator when described by the same scale, and standardized mean difference (SMD) was used as the effect indicator when described by different scales. Point estimates and 95%CI were given for each effect size. Because of the large heterogeneity of clinical studies, random-effects models were used for the meta-analysis. The heterogeneity was analyzed using the Q test and the *I*^2^ statistic, and *I*^2^ > 50% or *p*<0.05 indicated that the included studies were significantly heterogeneous. To explore the possible sources of heterogeneity, subgroup analyses were conducted based on time points of cognitive function assessment and types of included studies. Considering that the number of included studies for each outcome index was less than 10, no publication bias test was performed in this meta-analysis.

## 3 Results

### 3.1 Literature search

**Figure 1:**
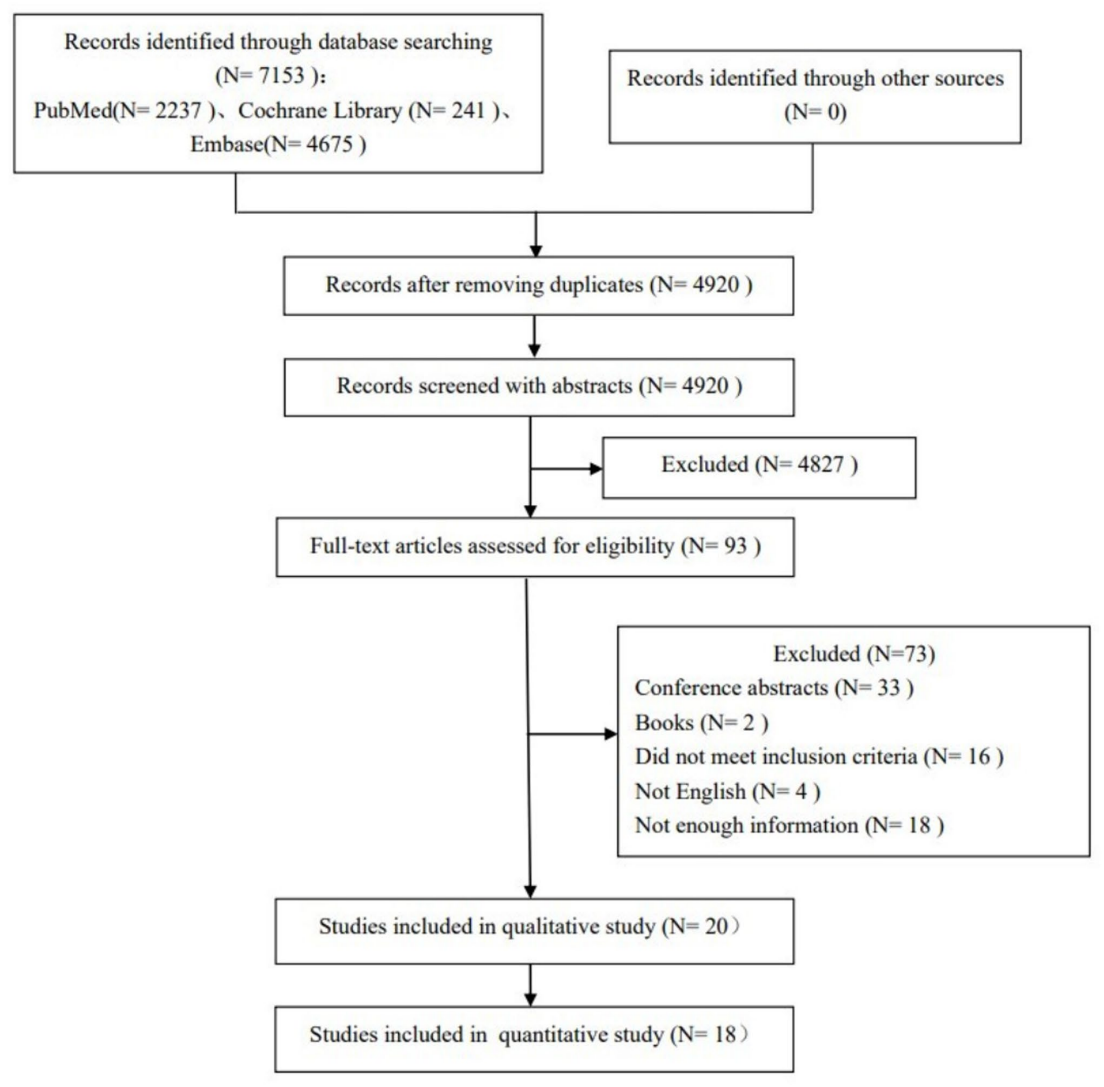
PRISMA diagram of eligible studies.

The preliminary search obtained 7,153 relevant studies, and 2,233 duplicate published studies were removed. After reading the title and abstract to exclude 4,827 studies that did not meet the inclusion criteria, the full text was read for re-screening. Finally, 20 clinical studies with a total of 704 epilepsy patients were included. The specific literature screening process and results are shown in Figure 1.

### 3.2 Study characteristics

A total of 20 studies were included, containing 6 randomized controlled studies and 14 non-randomized controlled studies (before-after study in the same patient). The 704 epilepsy patients ranged in age from 5 to 58 years. All patients were treated with invasive left cervical VNS. Each of the 20 studies included at least one cognitive function dimension. Outcome measures were measured from immediately after vagus nerve stimulation to more than 2 years after follow-up (specific details in Table 1).

**Table 1:** Trial characteristic.

					Experimental group	Control group		
Study	Country	Design	Epilepsy classification	Age group	Sample size	Female (%)	Age (year)	Epilepsy duration (year)	In/Non-in	Stimulation site	Pulse width (μs)	Frequency (Hz)	Amplitude (mA)	On-time/Off-time (s)	Duration (min)	Sample size	Female (%)	Age (year)	Epilepsy duration (year)	In/Non-in	Stimulation site	Pulse width (μs)	Frequency (Hz)	Amplitude (mA)	On-time/Off-time (s)	Duration (min)	Outcome	Measurement timepoint (month)
Hallböök et al. (7)	Sweden	Non-RCT	Therapy resistant epilepsy	Children	15	33.33%	11.33 ± 3.60	8.08 ± 2.25	In	Left neck	500	30	0.25 ~ (1–1.5)	30/300	NA	NA	NA	NA	NA	NA	NA	NA	NA	NA	NA	NA	Seizure frequency and severity, cognition, QOL, behavior, mood, depression, Side effects	3, 9
Tong et al. (15)	China	Non-RCT	Drug-resistant epilepsy (DRE) induced by tuberous sclerosis complex (TSC)	Children	6	33.33%	9.95 ± 4.46	3.29 ± 3.17	In	Left neck	250	30	0.5 ~ (1.25–1.5)	30/300	NA	NA	NA	NA	NA	NA	NA	NA	NA	NA	NA	NA	Seizure frequency, cognition, side effects	12
Achinivu et al. (16)	UK	Non-RCT	Treatment-resistant epilepsy	Adult	7	42.86%	39.29 ± 13.20	33.71 ± 15.32	In	Left neck	500	30	0.25 ~ (1–1.5)	30/300	NA	NA	NA	NA	NA	NA	NA	NA	NA	NA	NA	NA	Seizure frequency, cognition	12
Alonso-Vanegas et al. (17)	Mexico	Non-RCT	Refractory epilepsy (partial epilepsy 18, generalized epilepsy 17)	Adult, children	35	42.86%	23.3 ± 11.89	17.46 ± 10.53	In	Left neck	406.25 ± 62.5	24.22 ± 5	1.13 ± 0.69	30/180	NA	NA	NA	NA	NA	NA	NA	NA	NA	NA	NA	NA	Seizure frequency, cognition	35.6 ± 13.25
Danielsson et al. (18)	Sweden	Non-RCT	Drug-resistant epilepsy	Children	7	14.29%	13.50 ± 5.16	9.20 ± 3.00	In	Left neck	500	30	1.29 ± 0.34	30/180	NA	NA	NA	NA	NA	NA	NA	NA	NA	NA	NA	NA	Seizure frequency, cognition, autistic symptoms and behavior, Side effects	24
Wang et al. (19)	China	Non-RCT	Refractory epilepsy	Children	20	45.00%	11 ± 3.3	4.24 ± 3.23	In	Left neck	NA	NA	NA	NA	NA	NA	NA	NA	NA	NA	NA	NA	NA	NA	NA	NA	Seizure control, cognition	3, 6, 12
Tsai et al. (20)	China	Non-RCT	Refractory epilepsy	Children	37	48.60%	18 (<12), 19 (12–18)	NA	In	Left neck	500	30	0.25 ~ (1–1.5)	30/300	NA	NA	NA	NA	NA	NA	NA	NA	NA	NA	NA	NA	Seizure frequency, cognition, psychosocial adjustment	12
Soleman et al. (21)	Israel	Non-RCT	Drug-resistant epilepsy	Children	45	51.10%	11.16 ± 15.38	NA	In	Left neck	NA	NA	NA	NA	NA	NA	NA	NA	NA	NA	NA	NA	NA	NA	NA	NA	Seizure control, cognition, QOL	72.3 ± 39.8
Hoppe et al. (22)	Germany	Non-RCT	Pharmacoresistant complex–partial seizures	Adult	36	27.78%	33.6 ± 9.8	NA	In	Left neck	500	30	1.25(0.5–2)	30/300	NA	NA	NA	NA	NA	NA	NA	NA	NA	NA	NA	NA	Seizure frequency, cognition, neuropsychological	8 ± 2.8
Majoie et al. (23)	Netherlands	Non-RCT	Therapy-resistant epilepsy diagnosed as Lennox–gastaut syndrome	Children	16	18.75%	11.05(6–17)	7.9(4–14.3)	In	Left neck	500	30	0.25(0.5–2)	30/300	NA	NA	NA	NA	NA	NA	NA	NA	NA	NA	NA	NA	Seizure frequency and severity, cognition, neuropsychological, side effects	6 ~ 12
Tsai et al. (24)	China	Non-RCT	Medically refractory epilepsy	Chilidren, adult	105	56.20%	35 (33.3%) were aged 6–12 years, 33 (31.5%) aged 12–18 years and 12 (11.4%) aged >18 years	NA	In	Left neck	500	30	0.25 ~ 1.5	30/300	NA	NA	NA	NA	NA	NA	NA	NA	NA	NA	NA	NA	Seizure frequency, psychologically, cognition	3, 12
Clarke et al. (25)	Canada	Non-RCT	Intractable complex partial seizures	Adult	6	16.67%	33 ± 9.17	>20	In	Left neck	500/130	30/1	NA	NA	NA	NA	NA	NA	NA	NA	NA	NA	NA	NA	NA	NA	Cognition	28
Majoie et al. (26)	Netherlands	Non-RCT	Malignant childhood epilepsy resembling the Lennox–Gastaut syndrome	Children	19	21.05%	10.8(5.9–18.8)	NA	In	Left neck	500	30	0.25 ~ (1.5–2)	30/300	NA	NA	NA	NA	NA	NA	NA	NA	NA	NA	NA	NA	Cognition, QOL	6, 12, 18, 24
McGlone et al. (27)	Canada	Non-RCT	Medically uncontrollable complex partial seizures	Adult	16	43.75%	35 ± 8.0	NA	In	Left neck	500	30	2.0 ± 0.47	30/300	NA	9	66.67%	37 ± 6.7	NA	Medication controls(MC)	NA	NA	NA	NA	NA	NA	Quality of life (QOL), depressive affect, and memory	12
McGlone et al. (27)	Canada	Non-RCT	Medically uncontrollable complex partial seizures	Adult	16	43.75%	35 ± 8.0	NA	In	Left neck	500	30	2.0 ± 0.47	30/300	NA	10	60.00%	36 ± 12.7	NA	Cerebral resective surgery(RS)	NA	NA	NA	NA	NA	NA	Quality of life (QOL), depressive affect, and memory	12
Clarke et al. (28)	Canada	RCT	Intractable seizure activity	Adult	4	NA	NA	NA	In	Left neck	500	30	NA	NA	NA	4	NA	NA	NA	NA	NA	130	1	NA	NA	NA	Cognition	6
Dodrill and Morris (29)	USA	RCT	Uncontrolled partial seizures	Children, adult	78	53.00%	32.9 ± 10.9	21.93 ± 11.29	In	Left neck	500	30	Tolerated	30/300	NA	82	58.00%	35.3 ± 9.9	23.20 ± 11.19	In	Left neck	130	1	Perception	30/10800	NA	Cognition, QOL	3 ~ 4
Ghacibeh et al. (30)	USA	RCT	Medically intractable partial epilepsy	Adult	10	50.00%	46.70 ± 8.83	NA	In	Left neck	NA	NA	0.5	NA	30s (learning/recall)	10	50.00%	46.70 ± 8.83	NA	In	Left neck	NA	NA	0	NA	30s	Cognition	0
Sun et al. (31)	USA	RCT	Refractory epilepsy	Adult	20	40.00%	45 ± 13	NA	In	Left neck	250	30	1.5–1.75	30/48	6.13	20	40.00%	45 ± 13	NA	In	Left neck	NA	NA	0	NA	0	Cognition, EEG	0
Helmstaedter et al. (32)	Germany	RCT	Pharmakoresistant epilepsy	Adult	11	NA	31.9(18–42)	NA	In	Left neck	500	30	1.75(1–2.5)	270/300	4.5 min (stimulation was active during item presentation and recognition in all learning trials)	20	NA	19–45	NA	NA	NA	NA	NA	NA	NA	NA	Cognition	Before, during and after VNS
Clark et al. (33)	USA	RCT	Seizures	Adult	10	NA	NA	NA	In	Left neck	NA	NA	0.5	NA	NA	10	NA	NA	NA	In	Left neck	NA	NA	0	NA	NA	Cognition	0
Clark et al. (33)	USA	RCT	Seizures	Adult	10	NA	NA	NA	In	Left neck	NA	NA	0.75–1.5	NA	NA	10	NA	NA	NA	In	Left neck	NA	NA	0	NA	NA	Cognition	0

### 3.3 Bias and quality assessment

**Figure 2:**
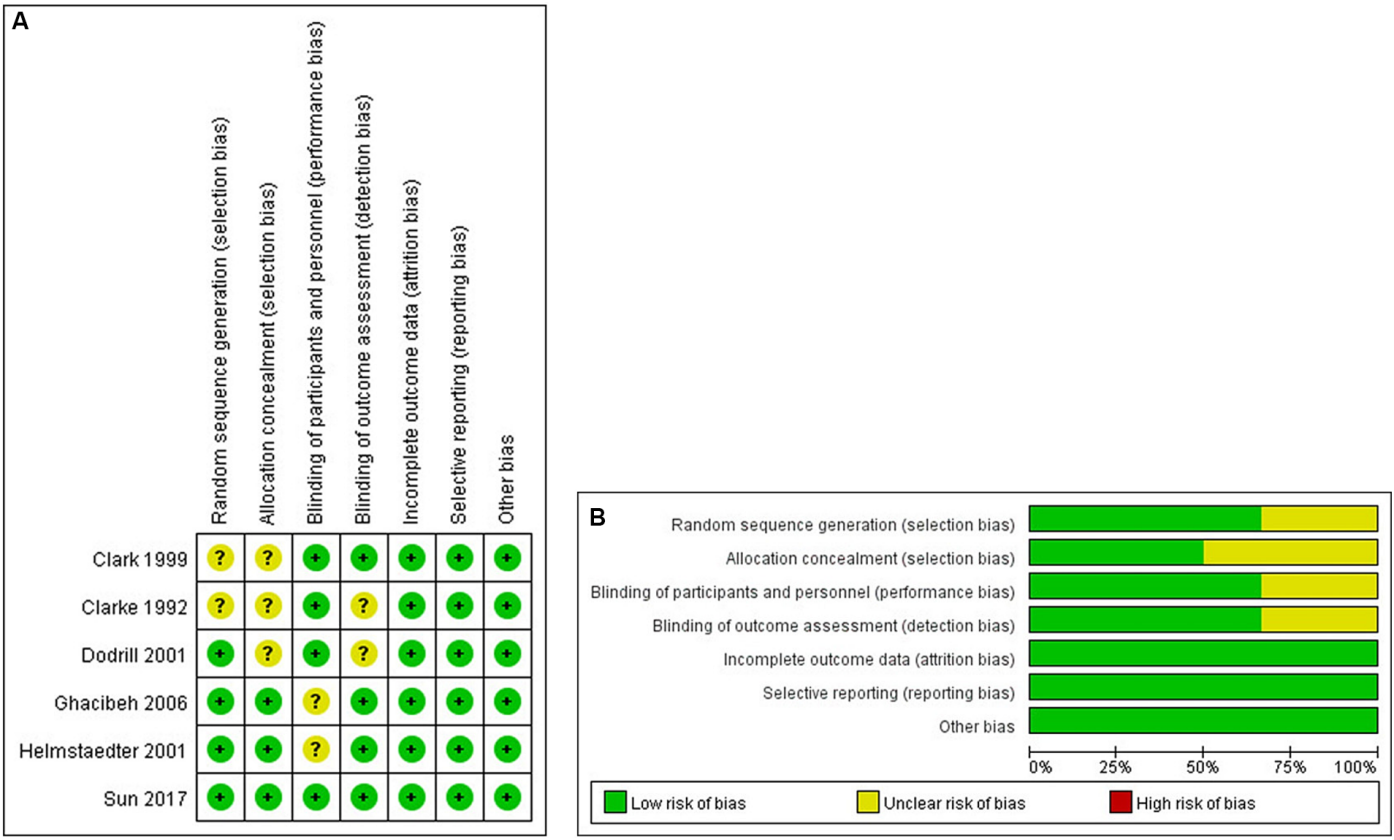
**(A,B)** Risk of bias summary and graph in RCTs.

Randomized controlled studies were assessed using the Cochrane Collaboration’s tool (Figures 2A,B). Non-randomized controlled studies were assessed using the MINORS with scores ranging from 12 to 20 (Table 2).

**Table 2:** Risk of bias and quality assessment of non-RCTs (MINORS).

Study	1	2	3	4	5	6	7	8	9	10	11	12	Total
Hallböök et al. (7)	2	2	2	2	1	2	2	0	NA	NA	NA	NA	13
Tong et al. (15)	2	2	2	2	0	2	2	0	NA	NA	NA	NA	12
Achinivu et al. (16)	2	2	2	2	2	2	2	0	NA	NA	NA	NA	14
Alonso-Vanegas et al. (17)	2	2	2	2	2	2	2	0	NA	NA	NA	NA	14
Danielsson et al. (18)	2	2	2	2	1	2	2	0	NA	NA	NA	NA	13
Wang et al. (19)	2	2	2	2	2	2	2	0	NA	NA	NA	NA	14
Tsai et al. (20)	2	2	2	2	1	2	2	0	NA	NA	NA	NA	13
Soleman et al. (21)	2	2	2	2	2	2	2	0	NA	NA	NA	NA	14
Hoppe et al. (22)	2	2	2	2	2	2	2	0	NA	NA	NA	NA	14
Majoie et al. (23)	2	2	2	2	2	2	2	0	NA	NA	NA	NA	14
Tsai et al. (24)	2	2	2	2	1	2	2	0	NA	NA	NA	NA	13
Clarke et al. (25)	2	2	2	2	0	2	2	0	NA	NA	NA	NA	12
Majoie et al. (26)	2	2	2	2	2	2	2	0	NA	NA	NA	NA	14
McGlone et al. (27)	2	2	2	2	1	2	2	0	2	2	1	2	20

### 3.4 Primary outcomes

#### 3.4.1 Overall cognitive performance

**Figure 3:**
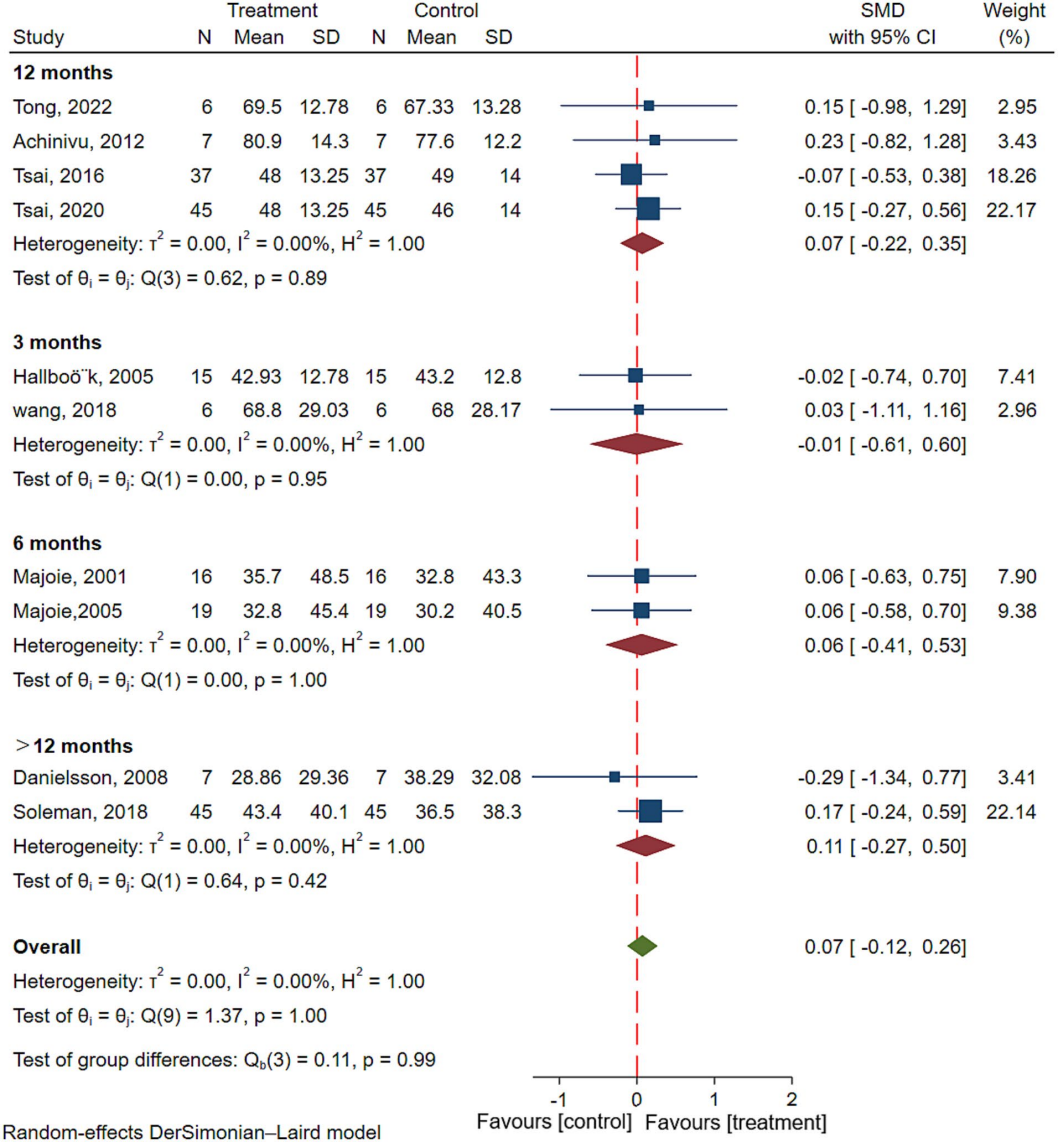
Forest plot showing the SMD and 95% CI of differences in overall cognitive performance between the VNS group and control group (the negative effect favors the control group, and the positive effect favors the VNS group).

Ten studies reported overall cognitive performance (Adult intelligence level or child development level) after VNS in patients with epilepsy, involving 200 patients with epilepsy (7, 15, 16, 18–21, 23, 24, 26). The results of meta-analysis showed that there was no significant improvement in overall cognitive performance in patients with epilepsy after VNS treatment (SMD = 0.07; 95% CI: −0.12 to 0.26; *I*^2^ = 0.00%) (Figure 3). To explore the effect of duration of VNS on overall cognitive performance, Subgroup analyses were performed. Results showed that there was no significant improvement in overall cognitive performance in patients with epilepsy after either 3 (SMD = 0.07; 95% CI: −0.12 to 0.26; *I*^2^ = 0.00%), 6 (SMD = 0.07; 95% CI: −0.12 to 0.26; *I*^2^ = 0.00%), 12 (SMD = 0.07; 95% CI: −0.12 to 0.26; *I*^2^ = 0.00%) or >12 (SMD = 0.07; 95% CI: −0.12 to 0.26; *I*^2^ = 0.00%) months of VNS treatment (Figure 3).

#### 3.4.2 Executive function

**Figure 4:**
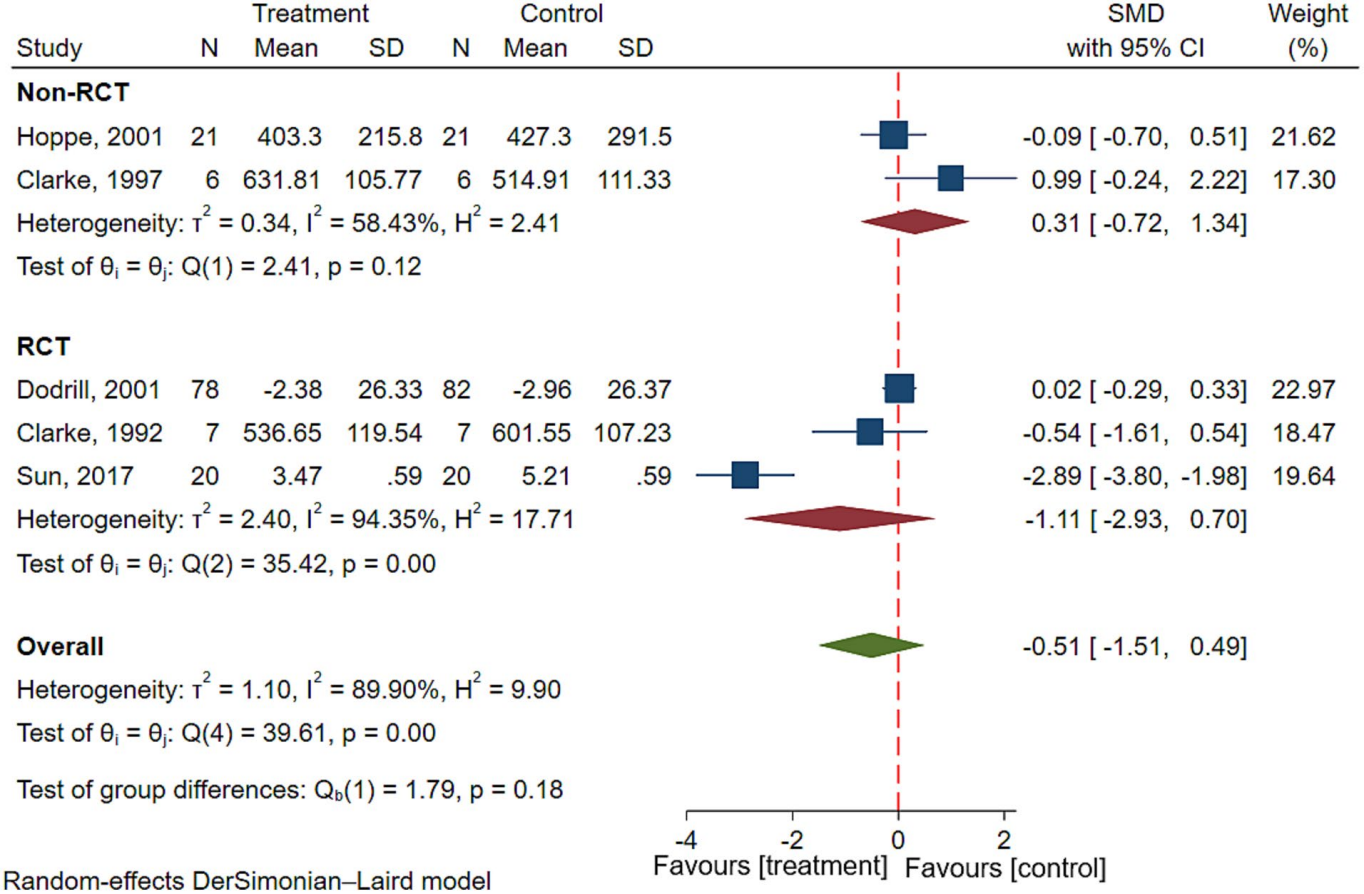
Forest plot showing the SMD and 95% CI of differences in executive function between the VNS group and control group. (the negative effect favors the VNS group, and the positive effect favors the control group).

Five studies reported executive function in patients with epilepsy, involving a total of 122 patients with epilepsy (22, 25, 28, 29, 31). Results of the meta-analysis showed there was no significant difference in executive function before and after VNS treatment in patients with epilepsy (SMD = −0.51; 95% CI: −1.51 to 0.49; *I*^2^ = 89.90%) (Figure 4). To explore the effect of types of studies on outcomes, Subgroup analyses were performed. Results showed that there was no significant improvement in executive function in patients with epilepsy in both RCTs (SMD = −1.11; 95% CI: −2.93 to 0.70; *I*^2^ = 94.35%) or non-RCTs (SMD = 0.31; 95% CI: −0.72 to 1.34; *I*^2^ = 58.43%) (Figure 4).

#### 3.4.3 Attention

**Figure 5:**
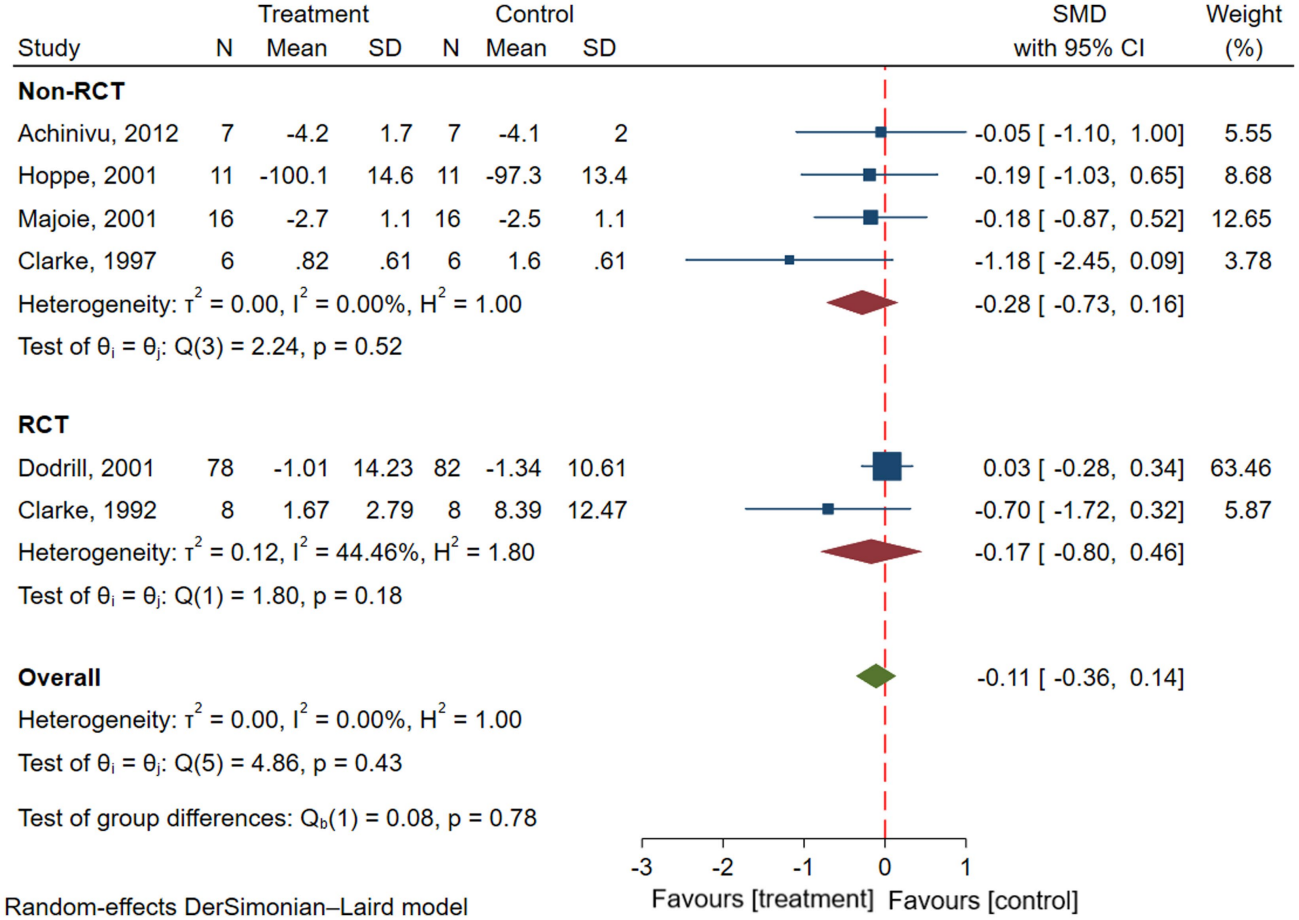
Forest plot showing the SMD and 95% CI of differences in attention between the VNS group and control group (the negative effect favors the VNS group, and the positive effect favors the control group).

Six studies reported attention in patients with epilepsy, involving a total of 146 patients with epilepsy (16, 22, 23, 25, 28, 29). Results of the meta-analysis showed there was no significant difference in attention before and after VNS treatment in patients with epilepsy (SMD = −0.11; 95% CI: −0.36 to 0.14; *I*^2^ = 0.00%) (Figure 5). To explore the effect of types of studies on outcomes, Subgroup analyses were performed. Results showed that there was no significant improvement in attention in patients with epilepsy in both RCTs (SMD = −0.17; 95% CI: −0.80 to 0.46; *I*^2^ = 44.46%) or non-RCTs (SMD = −0.28; 95% CI: −0.73 to 0.16; *I*^2^ = 0.00%) (Figure 5).

#### 3.4.4 Memory

**Figure 6:**
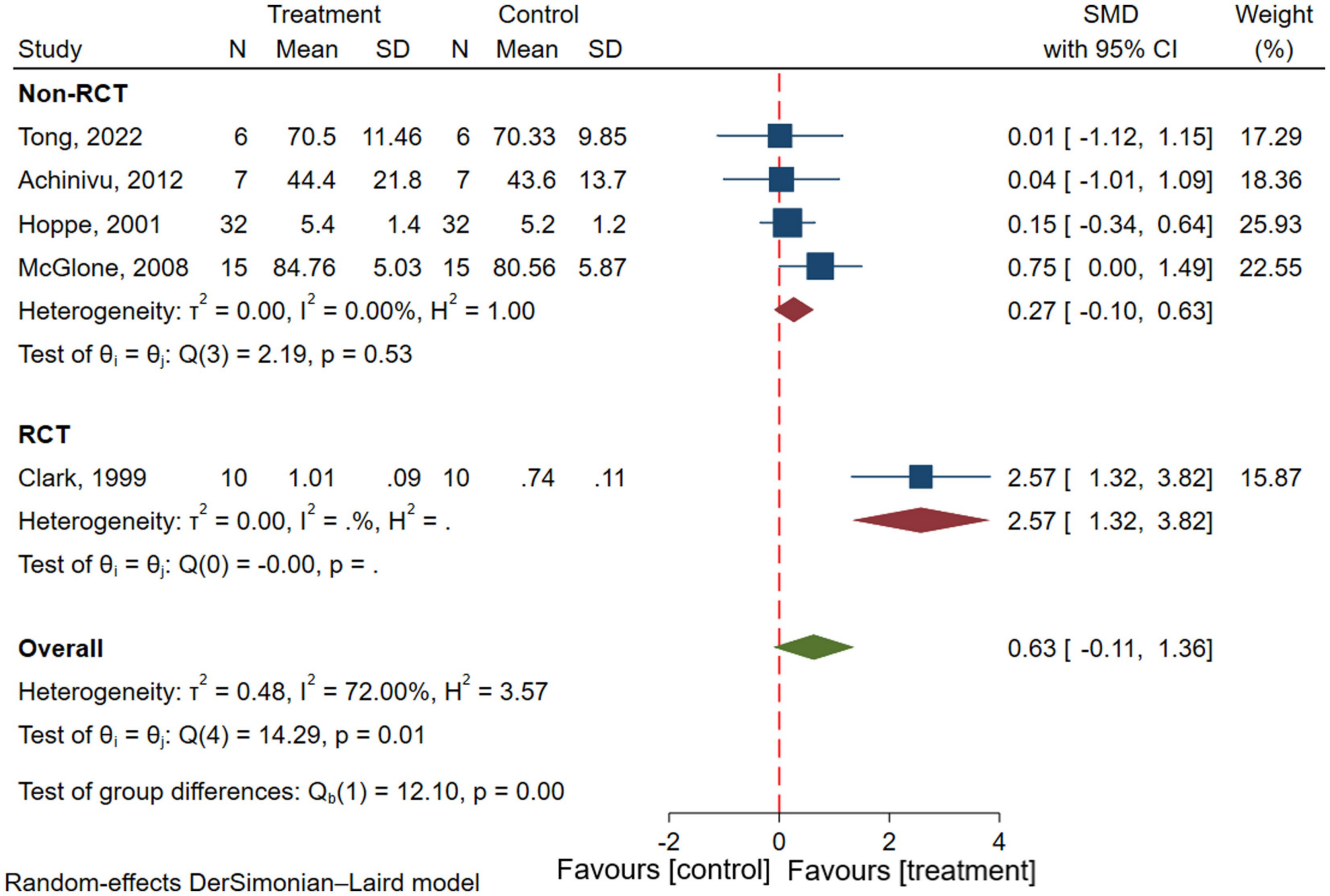
Forest plot showing the SMD and 95% CI of differences in memory between the VNS group and control group (the negative effect favors the control group, and the positive effect favors the VNS group).

Five studies reported memory in patients with epilepsy, involving a total of 70 patients with epilepsy (15, 16, 22, 27, 33). Results of the meta-analysis showed there was no significant difference in memory before and after VNS treatment in patients with epilepsy (SMD = 0.63; 95% CI: −1.11 to 1.36; *I*^2^ = 72.0%) (Figure 6). To explore the effect of types of studies on outcomes, Subgroup analyses were performed. Results showed that there was no significant improvement in memory in patients with epilepsy in non-RCTs (SMD = 0.27; 95% CI: −0.10 to 0.63; *I*^2^ = 0.00%) (Figure 6). But there was significant improvement in memory in patients with epilepsy in RCTs (SMD = 2.57; 95% CI: 1.32–3.82) (Figure 6).

### 3.5 Secondary outcomes

#### 3.5.1 Seizure frequency

Nine studies reported seizure frequency in patients with epilepsy, involving a total of 204 patients (7, 15–18, 20–23). Results of the meta-analysis showed that a significant decrease in seizure frequency after VNS, compared to pre-treatment (SMD = −0.32; 95% CI: −0.53 to −0.12; *p* < 0.001; *I*^2^ = 7.17%) (Table 3).

**Table 3:** Statistical values for secondary outcomes.

Outcomes	SMD	95%CI	*Z*	*P*	*I* ^2^
Seizure frequency	−0.32	(−0.53, −0.12)	−3.11	<0.001	7.17%
Mood	−0.59	(−1.13, −0.04)	−2.12	0.03	64.6%
QOL	0.50	(0.04, 0.95)	2.15	0.03	73.65%

#### 3.5.2 Mood

Four studies reported mood in patients with epilepsy, involving a total of 204 patients with epilepsy (7, 20, 26, 27). Results showed that compared with pre-treatment, the mood of patients with epilepsy after VNS treatment were significantly improved (SMD = −0.59; 95% CI: −1.13 to −0.04; *p* = 0.03; *I*^2^ = 64.6%) (Table 3).

#### 3.5.3 QOL

Six studies reported QOL in patients with epilepsy, involving a total of 204 patients (7, 17, 21, 23, 27, 29). Results of the meta-analysis showed that VNS treatment can significantly improve the quality of life of epilepsy patients (SMD = 0.50; 95% CI: 0.04 to 0.95; *p* = 0.03; *I*^2^ = 73.65%) (Table 3).

## 4 Discussion

As far as we know, this is the first meta-analysis exploring the effects of VNS on cognitive function in patients with epilepsy. Twenty clinical studies were included in this meta-analysis, including 14 non-randomized controlled trials and 6 randomized controlled trials, which included a total of 704 patients with epilepsy. All patients were diagnosed with refractory epilepsy, and all had a vagus nerve stimulator implanted in the left neck. Results showed that after VNS, the frequency of seizures in patients with refractory epilepsy was significantly reduced, and the mood and QOL were significantly improved compared with pre-treatment. This finding is consistent with the results of published systematic reviews and meta-analyses, and the efficacy of VNS in controlling seizures, improving depression or anxiety, and improving QOL was verified again (34–36). The meta-analysis showed that there were no significant differences in overall cognitive performance, executive function and attention before and after VNS treatment. However, there was a significant improvement in memory of patients with epilepsy in RCTs (33), but not in non-RCTs. However, only one randomized controlled study was included, and the reliability of the conclusion was poor.

The meta-analysis showed that there was no significant difference in the overall cognitive performance of epilepsy patients at 3, 6, 12, and > 12 months after VNS treatment compared with pre-treatment. However, it was observed that as the duration of VNS treatment increased, the overall cognitive performance in patients with epilepsy generally increased. This may be because VNS is a chronic electrical stimulation treatment, with small stimulation intensity and short on/off time, so its therapeutic effect is slow, and a longer follow-up is needed to explore its effects on cognitive function. A meta-analysis of the effects of transcutaneous-ear VNS on cognitive function in healthy individuals published in 2021, which included six high-quality RCTs, showed that transcutaneous-ear VNS significantly improved executive function in healthy individuals (37). However, the results of the meta-analysis showed that executive function and attention in epilepsy patients did not significantly improve after VNS treatment. This may be due to the pre-existing cognitive impairment in patients with epilepsy and it is difficult to improve cognitive outcomes for patients with cognitive impairment. Moreover, previous studies have shown that the parameters of VNS have a great influence on its efficacy, and the stimulation parameters in this meta-analysis, including stimulation site, stimulation time and so on, are different from those in the meta-analysis published in 2021. Regarding memory function, the results of the meta-analysis showed no significant improvement in memory function in patients with epilepsy before and after VNS. Although there was no statistically significant difference in the quantitative analysis, two high-quality RCTs published in 2001 and 2006 reported that VNS during the memory storage phase did not improve memory function in patients with epilepsy, whereas VNS during the memory recollection phase significantly improved memory function and chronic VNS significantly enhanced memory function compared with acute VNS (30, 32). Chronic VNS significantly enhanced memory function compared with acute vagus nerve stimulation, and the intensity of stimulation was preferable to moderate intensity. Therefore, more original clinical studies with large samples, rigorous design, and high quality are needed to clarify the effects of VNS on memory function in epilepsy patients.

So far, the stimulation of the left vagus nerve to control seizures has been studied for more than 100 years, and in 1997, it was officially approved by the United States Food and Drug Administration as a treatment for patients with refractory epilepsy over the age of 12 (38). Numerous studies have shown that VNS is effective in reducing the frequency of seizures in adolescent and adult patients (39–42). Although VNS requires invasive surgical procedures, the discomfort and side effects are mild and transient. The most common side effects are hoarseness, throat tingling and coughing, which are usually mild and disappear within a few weeks. As research has intensified in recent years, it has been observed in some studies that VNS has good efficacy not only for seizure control, but even for cognitive function in epileptic patients (10, 43, 44). The mechanism of action is not yet fully understood, but one widely accepted view is that long-term regular VNS can affect the corresponding neurological functional areas in an upward direction (45). After passing through the nucleus tractus solitarius and the reticular system of the medulla oblongata, electrical stimulation is transmitted to various brain subdivisions through the nuclei of the thalamus and the limbic system, thereby suppressing seizures. In addition to the cortical facilitation of cognitive functions, the thalamic nuclei and limbic system are widely recognized as being relevant to higher cognitive functions. Important components of the limbic system include the hippocampal structures, the para-hippocampal gyrus and the internal olfactory area, the dentate gyrus, the cingulate gyrus, the papillae, and the amygdala. These structures are interconnected through the Papez Ring and have extensive connections with other brain structures (neocortex, thalamus, brainstem), so the limbic system serves to enable information exchange between the midbrain, mesencephalon, and neocortical structures. Studies have shown that stimulation of the hippocampal region can have a dramatic effect on memory function and executive function (45). The impact of continuous and regular stimulation of the limbic system and cerebral cortex by the VNS on cognitive function is immeasurable. Another emerging mechanism of VNS in improving cognitive function in patients with epilepsy is that pairing VNS with a cognitive task amplifies task-induced cerebral blood flow (CBF) in the prefrontal cortex and enhances the plasticity of the specific task in the cortex, leading to improvement in cognitive function in patients with epilepsy (46). Kunii et al. study verified this hypothesis, which observed in patients with epilepsy that CBF was increased only when VNS was paired with a cognitive task and that VNS alone did not alter CBF (47). However, most of the studies included in this meta-analysis used VNS alone, not paired with a cognitive task, which may be one of the reasons for the negative results of this meta-analysis. Kunii et al. study also suggests that both the degree and rate of increase in CBF are positively correlated with the stimulus intensity of VNS (47). The greater the stimulus intensity of VNS, the greater and faster the increase in CBF. It shows that precise timing of VNS and the task and a high enough VNS dose are necessary for efficient coupling between VNS and rehabilitation. This prompted that future clinical studies on VNS improving cognitive function in patients should pay attention to pairing VNS with specific cognitive tasks and to adjusting the intensity of VNS stimulation.

Limitations of this systematic review and meta-analysis: (1) the included studies showed significant heterogeneity in several aspects: differences in study populations and VNS parameters, with high heterogeneity in executive function and memory outcomes; (2) the included studies were published in English only, which may indicate the presence of language and publication bias; (3) most of the studies included in this meta-analysis were non-randomized controlled trials, the overall quality of the included studies was low.

In conclusion, the results of this meta-analysis suggest that there are insufficient data to demonstrate significant improvements in overall cognitive performance, executive function, attention, and memory in patients with epilepsy, but VNS may significantly improve seizure frequency, mood, and quality of life in patients with epilepsy. In the future, more high-quality, long follow-up, large-scale, multicenter randomized controlled trials that strictly follow the CONSORT guidelines are needed in the future to assess the effects of VNS on cognitive function in patients with epilepsy.

## Data availability statement

The original contributions presented in the study are included in the article/Supplementary material, further inquiries can be directed to the corresponding author.

## Author contributions

YK: Conceptualization, Data curation, Software, Writing – original draft, Writing – review & editing. KZ: Methodology, Visualization, Writing – review & editing. DZ: Methodology, Project administration, Resources, Writing – review & editing. FL: Data curation, Formal analysis, Writing – review & editing. XL: Investigation, Methodology, Writing – review & editing. YW: Project administration, Resources, Software, Writing – review & editing. ZJ: Formal analysis, Writing – review & editing. WW: Conceptualization, Supervision, Validation, Visualization, Writing – review & editing.
