# Supplementary material for: Effects of vagus nerve stimulation on cognitive function in patients with epilepsy: a systematic review and meta-analysis
Source: Front Neurol. 2024 Feb 9;15:1332882. doi: 10.3389/fneur.2024.1332882 (PMC10884318; doi:10.3389/fneur.2024.1332882)
Supplement: Supplementary file 1 [file Data_Sheet_1.docx]

**Supplementary Materials**

1. **Supplementary Table**

**Table S1. Search strategy**

| **Database** | **#** | **Search strategy** | **Results** |
| --- | --- | --- | --- |
| PubMed | 1 | Epilepsy[MeSH] | 122,737 |
|  | 2 | Seizures[MeSH] | 71,071 |
|  | 3 | epilep*[Title/Abstract] OR seizure*[Title/Abstract] OR convuls*[Title/Abstract] | 238,770 |
|  | 4 | #1 OR #2 OR #3 | 266,736 |
|  | 5 | Vagus Nerve Stimulation[MeSH] | 2,189 |
|  | 6 | (vagus[Title/Abstract] OR vagal[Title/Abstract]) AND Stimul*[Title/Abstract] | 14,127 |
|  | 7 | #5 OR #6 | 14,326 |
|  | 8 | #4 AND #7 | 2,237 |
|  | 1 | MeSH descriptor: [Epilepsy] explode all trees | 2,604 |
| Cochrane library | 2 | MeSH descriptor: [Seizures] explode all trees | 1,247 |
|  | 3 | (epilep* OR seizure* OR convuls*):ti,ab,kw | 14,668 |
|  | 4 | #1 OR #2 OR #3 | 14,702 |
|  | 5 | MeSH descriptor: [Vagus Nerve Stimulation] explode all trees | 128 |
|  | 6 | ((vagus OR vagal) AND Stimul*):ti,ab,kw | 1,449 |
|  | 7 | #5 OR #6 | 1,449 |
|  | 8 | #4 AND #7 | 241 |
| EMBASE | 1 | 'epilepsy'/exp | 281,877 |
|  | 2 | 'seizure'/exp | 176,698 |
|  | 3 | epilep*:ab,ti OR seizure*:ab,ti OR convuls*:ab,ti | 342,802 |
|  | 4 | #1 OR #2 OR #3 | 453,898 |
|  | 5 | 'vagus nerve stimulation'/exp | 12,280 |
|  | 6 | (vagus:ab,ti OR vagal:ab,ti) AND stimul*:ab,ti | 18,387 |
|  | 7 | #5 OR #6 | 23,038 |
|  | 8 | #4 AND #7 | 4675 |

**Search date:** 2022/09/20

**Table S2. Cognitive measurement scales or methods for each study**

| **Cognitive domain** | **Study** | **Cognitive measurement scales or methods** |
| --- | --- | --- |
| Overall cognitive performance | Tong, 2022 | The Wechsler Preschool and Primary Scale of Intelligence-Fourth Edition (aged 4–6 years),  the Wechsler intelligence scale for children-Fourth Edition (aged 6–16 years). |
|  | Achinivu, 2012 | Raven’s Standard Progressive Matrices |
|  | Tsai, 2016 | the Bayley Scales of Infant Development,  Wechsler Intelligence Scale for Children — IV. |
|  | Tsai, 2020 | the Wechsler Intelligence Scale(age＜12) |
|  | Hallboö¨k, 2005 | Bayley Scales of Infant Development（BSID）American version,  Wechsler Pre-school and Primary Scale of Intelligence (WPPSI-R) Swedish version,  Wechsler Intelligence Scales for Children（WISC-III）Swedish version. |
|  | wang, 2018 | Chinese edition of WISC‑IV |
|  | Majoie, 2001 | the Bayley Developmental Scale (with scores ranging from 2 to 30 months of mental age),  the McCarthy Developmental Scale (with scores ranging from 2.5 to 8,5 years of mental age),  the Wechsler Intelligence Scale for Children (with scores ranging from 6 to 17 years of mental age). |
|  | Majoie,2005 | the Dutch version of the Bayley Developmental Scale20 (with scores ranging from 2 to 30 months mental age),  the McCarthy Developmental Scale21 (with score ranging from 2(1/2) to 8(1/2) years mental age),  the WISC RN22 (with scores ranging from 6 to 17 years mental age). |
|  | Danielsson, 2008 | Griffiths’ Developmental Scales (mental age＜3),  Wechsler Preschool and Primary Scale of Intelligence (mental age3-7),  Wechsler Adult Intelligence Scale III (age＞16). |
|  | Soleman, 2018 | Pediatric Quality of Life-cognitive |
| Executive function | Hoppe, 2001 | Maze Test (Chapuis)-time |
|  | Clarke, 1997 | inhibition-time |
|  | Dodrill, 2001 | Stroop Test-interference-time |
|  | Clarke, 1992 | inhibition-time |
|  | Sun, 2017 | The Executive-RT test-error |
| Attention | Achinivu, 2012 | Cognitrone |
|  | Hoppe, 2001 | d2 cancellation test-standard score |
|  | Majoie, 2001 | scored by the test technician using observations made during the full day of assessment |
|  | Clarke, 1997 | reaction time-error |
|  | Dodrill, 2001 | Digit Cancellation-omitted |
|  | Clarke, 1992 | reaction time-error |
| Memory | Tong, 2022 | The Wechsler Preschool and Primary Scale of Intelligence-Fourth Edition (aged 4–6 years).  the Wechsler intelligence scale for children-Fourth Edition (aged 6–16 years) |
|  | Achinivu, 2012 | Continuous Visual Recognition Task |
|  | Hoppe, 2001 | digit span-forward |
|  | McGlone, 2008 | the Wechsler Memory Scale – III (WMS-III) |
|  | Clark, 1999 | Normalized mean word recognition score |

**Table S3. Mood or QOL scales for each study**

| **Secondary outcomes** | **Study** | **Scales** |
| --- | --- | --- |
| Mood | Hallboö¨k, 2005 | Dodrill Mood Analogue Scale scores |
|  | Tsai, 2016 | the Parental Stress Index (PSI) |
|  | Majoie,2005 | Mood (TVZ score) |
|  | McGlone, 2008 | The Geriatric Depression Scale |
| Quality of life  (QOL) | Hallboö¨k, 2005 | visual analogue scales |
|  | Soleman, 2018 | Pediatric Quality of Life |
|  | Vanegas, 2010 | The Quality of Life in Epilepsy-31 |
|  | Dodrill, 2001 | Quality of Life in Epilepsy-31 |
|  | Majoie, 2001 | scored by the test technician using observations made during the full day of assessment |
|  | McGlone, 2008 | The Quality of Life Inventory in Epilepsy-89 (QOLIE-89) |

1. **Supplementary Figures**


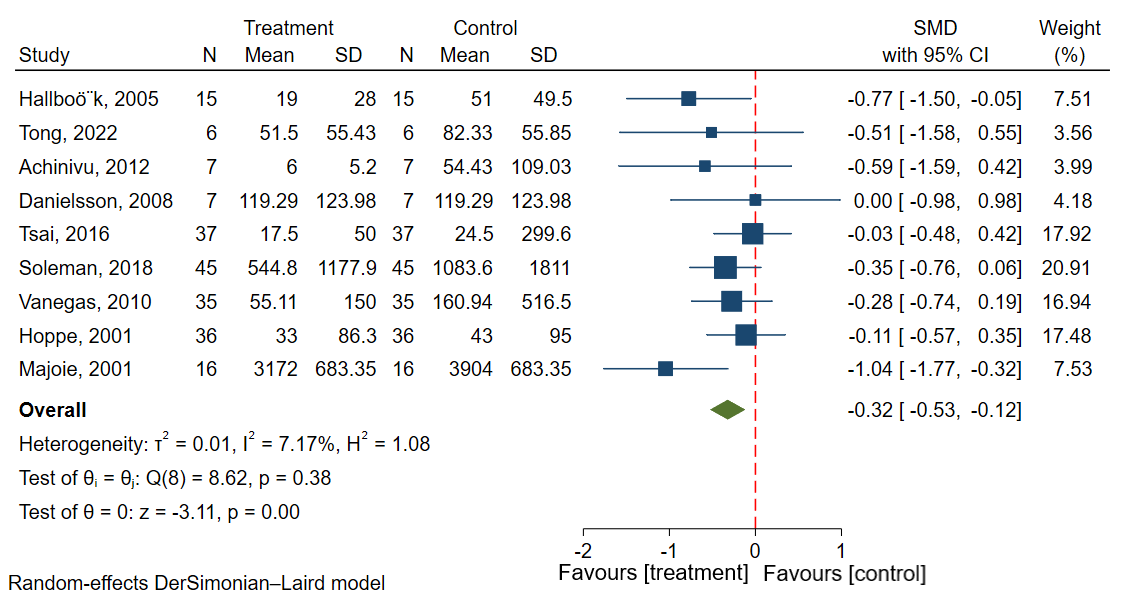


**Figure S1. Forest plot showing the SMD and 95% CI of differences in seizure frequency between the VNS group and control group. (the negative effect favours the VNS group, and the positive effect favours the control group).**

**
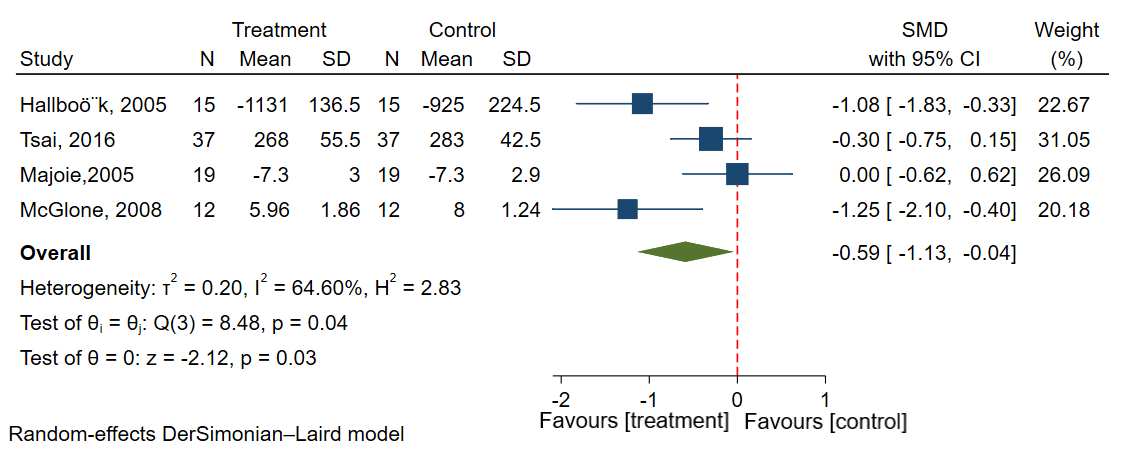
**

**Figure S2. Forest plots of mood. (the negative effect favours the VNS group, and the positive effect favours the control group).**

**
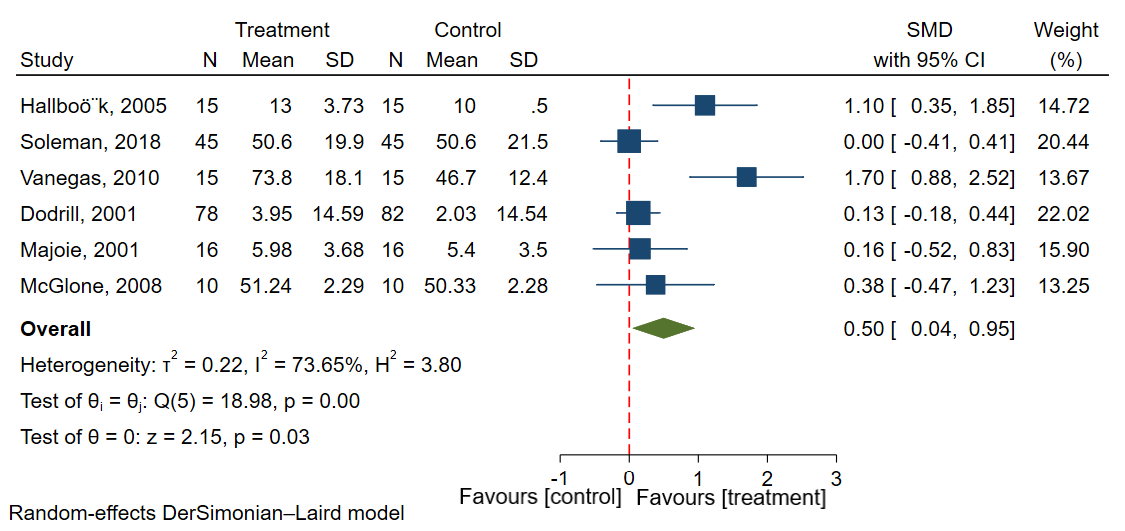
**

**Figure S3. Forest plots of quality of life (QOL). (the negative effect favours the control group, and the positive effect favours the VNS group).**
